# Vaccinia-related kinase 1 (VRK1) confers resistance to DNA-damaging agents in human breast cancer by affecting DNA damage response

**DOI:** 10.18632/oncotarget.1678

**Published:** 2014-01-17

**Authors:** Marcella Salzano, Marta Vázquez-Cedeira, Marta Sanz-García, Alberto Valbuena, Sandra Blanco, Isabel F. Fernández, Pedro A. Lazo

**Affiliations:** ^1^ Experimental Therapeutics and Translational Oncology Program, Instituto de Biología Molecular y Celular del Cáncer, Consejo Superior de Investigaciones Científicas (CSIC), Universidad de Salamanca, Salamanca, Spain; ^2^ Instituto de Investigación Biomédica de Salamanca (IBSAL), Hospital Universitario de Salamanca, Salamanca, Spain

**Keywords:** Breast cancer, VRK1, Radiation, doxorubicin, chemotherapy, DNA damage, 53BP1

## Abstract

Vaccinia-related kinase 1 (VRK1) belongs to a group of sixteen kinases associated to a poorer prognosis in human breast carcinomas, particularly in estrogen receptor positive cases based on gene expression arrays. In this work we have studied the potential molecular mechanism by which the VRK1 protein can contribute to a poorer prognosis in this disease. For this aim it was first analyzed by immunohistochemistry the VRK1 protein level in normal breast and in one hundred and thirty six cases of human breast cancer. The effect of VRK1 to protect against DNA damage was determined by studying the effect of its knockdown on the formation of DNA repair foci assembled on 53BP1 in response to treatment with ionizing radiation or doxorubicin in two breast cancer cell lines. VRK1 protein was detected in normal breast and in breast carcinomas at high levels in ER and PR positive tumors. VRK1 protein level was significantly lower in ERBB2 positive cases. Next, to identify a mechanism that can link VRK1 to poorer prognosis, VRK1 was knocked-down in two breast cancer cell lines that were treated with ionizing radiation or doxorubicin, both inducing DNA damage. Loss of VRK1 resulted in reduced formation of DNA-damage repair foci complexes assembled on the 53BP1 scaffold protein, and this effect was independent of damaging agent or cell type. This observation is consistent with detection of high VRK1 protein levels in ER and PR positive breast cancers. We conclude that VRK1 can contribute to make these tumors more resistant to DNA damage-based therapies, such as ionizing radiation or doxorubicin, which is consistent with its association to a poor prognosis in ER positive breast cancer. VRK1 is potential target kinase for development of new specific inhibitors which can facilitate sensitization to other treatments in combination therapies; or alternatively be used as a new cancer drugs.

## INTRODUCTION

Breast cancer represents one of the most frequent tumors in humans, and despite a significant improvement in survival resulting from an earlier diagnosis and new therapies; it still represents a major medical problem. Several gaps need answers to improve management of these tumors, and identification of markers for radiosensitivity and chemosensitivity is one of them [1]. There are three major types of breast cancer based on the expression of estrogen and progesterone receptors and ERBB2, a member of the EGF receptor family. The combination of their expression levels identifies the major groups susceptible to current treatments, directed either to hormone receptors or the ERBB2 receptor. Also, there is still a smaller, but very important group that includes cases that are negative for these three markers, known as triple negative and this type of tumors still do not have a suitable treatment [2].

Gene expression analysis has led to a wealth of information regarding tumor characterization, and permitted the identification of different tumor types based on gene expression patterns. In human breast cancer the analysis of gene expression has led to the identification of several kinases as potential prognostic markers [3, 4]. These studies identified the VRK1 kinase as a marker for a subgroup with a poorer prognosis within the estrogen receptor positive cases. This gene expression pattern is mostly reproduced in human breast cancer cell lines [5]. Moreover, the analysis of the human kinome in breast cancer cases led to the identification of a gene expression signature containing sixteen kinases that was associated with a poorer prognosis, and VRK1 was also present in this group [6]. This association was mainly detected in luminal tumors, suggesting their potential use as marker or therapeutic targets in this subgroup of breast cancers [6].

VRK1 is a nucleosomal, or chromatin, kinase that directly and stably interacts with different chromatin proteins such as histone H3[7], macroH2A1.2 [8], and HP1 [7]. In addition VRK1 also interacts and phosphorylates several transcription factors including c-Jun [9], ATF2 [10], CREB [11] and p53[12-14], all of them involved in oncogenesis. The VRK1 gene appeared late in evolution and its gene complexity parallels that of the p53 gene family [15], suggesting that this kinases play a coordinating role as organisms become more complex. VRK1 has been associated with control of cell cycle progression where it plays different roles [16]. VRK1 is required for exit G0 cell cycle phase and entry in G1 [17], behaving as an early gene like *MYC* and *FOS*, but later is also required for chromatin condensation by phosphorylation of histone H3 [7, 18], nuclear envelope organization [19] and Golgi fragmentation late in mitosis [20]. In addition VRK1 has been implicated in responses to DNA damage induced by UV-light [14] and by ionizing radiation [21]. This latter effect is mediated by regulation of DNA repair foci assembled on the 53BP1 scaffold protein [22]. Reduction of VRK1 levels impaired 53BP1 foci formation and also resulted in defective activation of the ATM-CHK2 pathway [21]. Moreover, in human cancer, VRK1 has been associated to the proliferation phenotype and is co-expressed with Ki67 in head and neck squamous cell carcinoma [23]. Also VRK1 is expressed at high levels, correlating with Ki67 and p63 in non-small lung cancer [24] and high-grade astrocytomas [25]. These effects of VRK1 indicated that it might contribute to tumor prognosis by modulation of tumor proliferation and cellular responses to DNA-damage based treatments.

In this work we have validated that VRK1 protein is present at significantly higher levels in breast carcinomas that are positive for hormone receptors (estrogen and progesterone). Moreover, we provided evidence about VRK1 biological significance in human breast cancer cell lines, since this kinase contributes to cell protection against DNA damage induced by therapy, and this function can be relevant for conferring a poorer prognosis to breast cancer cases.

## RESULTS

### Expression of VRK1 protein in normal human breast

**Figure 1:**
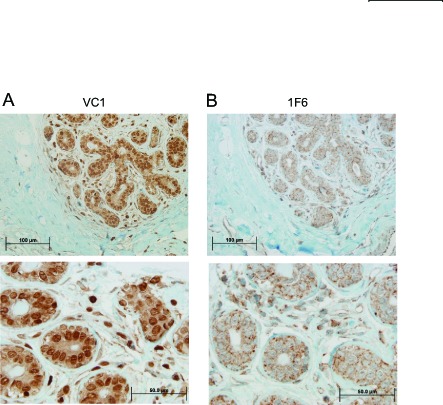
Expression of VRK1 in normal human breast tissue The VRK1 protein was determined with two different antibodies recognizing the two VRK1 intracellular populations. Two magnifications are shown. A. Detection of the main nuclear VRK1 population with a rabbit polyclonal antibody (VC1). B. Detection of cytosolic subpopulation of VRK1 using the 1F6 monoclonal antibody specific for this cytosolic subpopulation.

Initially it was determined the presence of VRK1 protein in normal mammary gland tissue by immunohistochemistry. In human mammary gland high level of nuclear VRK1 protein was detected mainly in cells located in the luminal side (Fig. 1A). However, all mammary epithelial cells expressed this protein (Fig. 1A). Also the expression of a minor cytoplasmic subpopulation was detected in cytosol (Fig. 1B) using the 1F6 mAb [20, 26]. This cytosolic subpopulation presented a similar level of expression in all epithelial mammary cells, independent of its location (Fig. 1B). Other cell types in mammary gland stroma presented a significantly lower level of cytosolic VRK1 protein.

### VRK1 correlation with ER, PR and ERBB2 in human breast cancer

**Figure 2:**
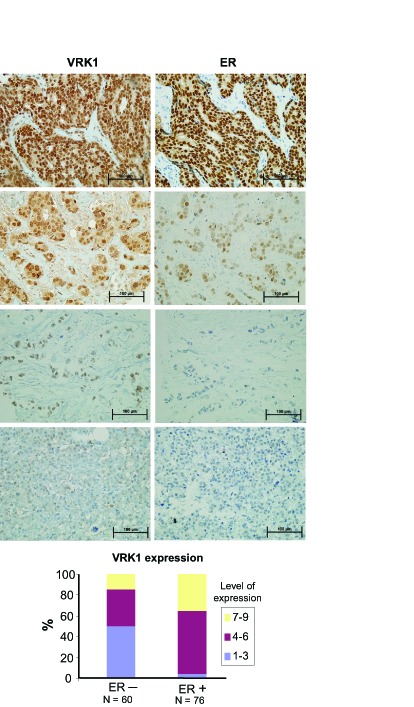
Expression of VRK1 and estrogen receptor in breast cancer Different levels of VRK1 expression as a function of the level of estrogen receptor. VRK1 was detected with rabbit polyclonal VC1antibody.In the graph at the bottom are shown the relatives levels of VRK1 as a function of ER positivity or negativity.

Different studies using RNA microarrays detected high levels of VRK1 expression in estrogen receptor positive breast cancer and at the same time the group with high VRK1 identified patients with a poorer prognosis [3, 4, 6, 27]. Based on this data we decided to study VRK1 protein expression in a panel of biopsies containing two groups of breast cancers, ER+/ERBB2- and ER-/ERBB2+. VRK1 positively correlated with estrogen and progesterone receptor positivity and inversely correlated with ERB2 positivity. Examples of these different levels of expression are shown for VRK1/ estrogen receptor (ER) (Fig.2) and VRK1/progesterone receptor (PR) (Fig. S1).The relative level of VRK1 expression as a function of estrogen receptor positivity is shown in Fig. 2 (bottom). VRK1 positively correlated (P<0.0001) with either ER or PR, and negatively correlated with ERBB2 (P<0.002). Also it was detected that VRK1 is downregulated in ERBB2 positive tumors (Fig. S2), a situation similar to that reported for VRK2 and ERBB2 [28, 29].

### VRK1 and VRK2 levels in human breast cancer cell lines

**Figure 3:**
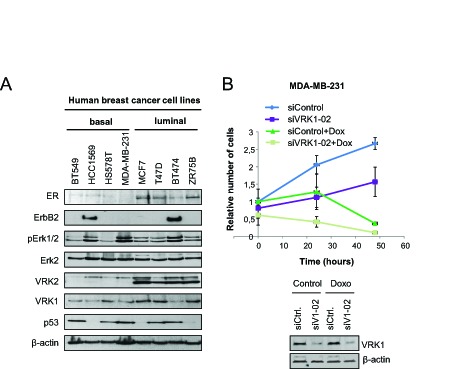
Expression of VRK1, VRK2, ER, ERBB2 and signaling proteins in a panel of breast cancer cell lines A. Expression of different proteins in eight breast cancer cell lines. B. Effect of VRK1 on cell growth. MDA-MB-231 cells were transfected with siVRK1-02 (SiV1-02) or siControl (siCtrl) using Lipofectamine2000. After 48 hours, cells were treated with Doxorubicin (1 µM) and cells were counted at 0 h, 24 h and 48 h. The mean of relative number of cells and the standard deviations were represented in the graph.

The expression of VRK1 and VRK2 as well as estrogen receptor and ERBB2 were determined in a panel of eight human breast cancer cell lines (Fig. 3A), including four of each type, luminal or basal [30]. Luminal type cell lines, which are positive for estrogen receptor, have in general a higher level of VRK1 than basal cell lines. Also in luminal cell lines the level of total VRK2 is also higher, in part due to expression of VRK2B isoform, but its significance is unknown. Also activation of MAPK associated in part to proliferation signaling was detected by the presence of phospho-Erk, whci is higher in the two cell lines expressing ERBB2 (HCC1569 and BT474). This is consistent with the negative correlation between ERBB2 and VRK1detected in immunohistochemistry, cell lines with higher level of ERBB2 showed lower VRK1 levels. Cell lines with mutated p53 are detected by its accumulation and are equally distributed among luminal and basal cell lines as expected [30]. We selected one luminal, MCF7 (ER positive), and one basal, MDA-MB-231(ER negative) cell lines to further characterize the consequences of manipulating their VRK1 levels. Since VRK1 has been associated to proliferation it was determined how knockdown of VRK1 affected cell number using MDA-MB-231, as well as the effect of treatment with doxorubicin. VRK1 knockdown reduced increases in cell number, that was even stronger in case of treatment with doxorubicin (Fig. 3B), as expected based on the known role of VRK1 in cell proliferation.

### VRK1 knockdown impaired formation of 53BP1 foci in response to DNA damage induced by ionizing radiation or doxorubicin.

**Figure 4:**
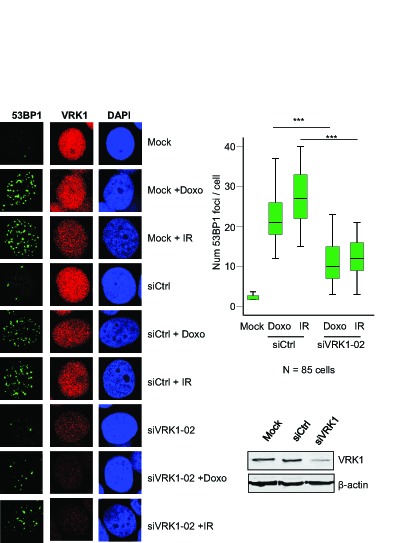
Effect of VRK1 knockdown on 53BP1 foci induced by ionizing radiation (IR) or doxorubicin in MCF7 breast cancer cells At the left is shown the immunofluorescence. The number of foci 53BP1 foci counted in seventy-five cells is shown in the graph. The image of the field is shown in Fig. S3. VRK1 was detected with rabbit polyclonal VC1antibody.

**Figure 5:**
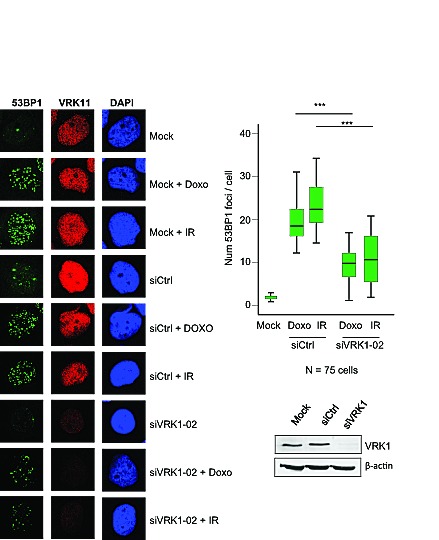
Effect of VRK1 knockdown on 53BP1 foci induced by ionizing radiation (IR) or doxorubicin in MDA-MB-231 breast cancer cells At the left is shown the immunofluorescence. The number of foci 53BP1 foci counted in eighty-five cells is shown in the graph. The image of the field is shown in Fig. S4. VRK1 was detected with rabbit polyclonal VC1antibody.

The level of VRK1 can regulate the ability of the cell to respond to DNA damage, which is a common mechanism to different types of therapy used in breast cancer. These therapies including ionizing radiation or doxorubicin, a topoisomerase inhibitor used in different combination protocols, are able to generate double-strand DNA breaks that will trigger protective DNA-damage response pathways. The level of VRK1 protein regulates the formation of 53BP1 DNA-repair foci induced by ionizing radiation in lung cancer cell lines [21]. Therefore it was studied if the level of VRK1 protein can affect the formation of 53BP1 DNA-repair foci in response to ionizing radiation or doxorubicin [31]. In this study two breast cancer cell lines were used MCF7, luminal [30], and MDA-MB-231, basal B [30]. Knockdown of endogenous VRK1 was performed with two different siVRK1 and the formation of 53BP1 foci, reflecting proper activation of DNA repair [32], was determined. The loss of VRK1 resulted in a significant reduction of the 53BP1 foci induced by either ionizing radiation or doxorubicin in MCF7 (Fig. 4) and MDA-MB-231 (Fig. 5) cells. These results indicated that high VRK1 levels confer resistance to DNA-damage based treatments by permitting activation of DNA repair pathways. Facilitating DNA repair in DNA-damage based treatments against cancer cells can contribute to a poorer prognosis of tumors with high levels of VRK1.

### DISCUSSION

Receptor positive breast cancer has high levels of VRK1, a situation that has also been reported in other tumors such as head and neck squamous cell carcinomas [23], non-small lung cancer [24] and high-grade astrocytomas [25]. In addition, high VRK1 levels have also been detected in tumors harboring p53 mutations and in which the VRK1-p53 downregulatory loop is defective [14, 33, 34].

The contribution of VRK1 to a poorer prognosis can be a consequence of VRK1 as a chromatin kinase [7, 8] since it affects cellular response to DNA damage and is required for the formation of 53BP1 foci in response to DNA double strand breaks induced by ionizing radiation [21]. High levels of VRK1 exert a protective effect against DNA damage induced either by ionizing radiation or chemotherapeutic drugs. ER positive tumors of the luminal type are characterized by an enhanced chemoresistance [31]. Thus, VRK1 high levels can confer a poorer prognosis because of their better resistance in tumors treated with DNA-damage based therapies. High VRK1 levels can also have an anti-tumoral effect by VRK1 specific phosphorylation of p53 in Thr18 converting it into a transcriptionally active p53 [12, 13, 34] that can trigger cell cycle arrest or induce death and subsequent elimination of tumor cells. But p53 levels are tightly regulated by induction of a double auto regulatory loop mediated by p53-dependent proteins, such as mdm2 that targets p53 for proteasomal degradation, and DRAM that results in elimination of VRK1 in the autophagic pathway [14]. However p53 is mutated in a very large number of cancers, and p53 mutants are unable to induce downregulation of p53[33] and VRK1[14], by different mechanisms. The consequence is that tumors harboring p53 mutations have higher levels of VRK1 by the inactivation of its downregulation, as has already been identified in lung cancer [24]. Thus, breast cancers with p53 mutations they will be even more resistant to DNA-damage based treatments because they will be unable to induce cell death.

Another role by which VRK1 contributes to a poorer tumor prognosis can be due to its role in proliferation [16]. VRK1 is regulated in cell cycle and its effect is mediated by the contribution to the coordination of several processes required for cell division, such as disassembly and reassembly of the nuclear membrane [19, 35], Golgi fragmentation late in mitosis [20] or chromatin condensation [7]. In experimental animal models, human breast cancer xenographs expressing high levels of VRK1 have a higher growth rate and invasive/dissemination potential [36]. Thus one contribution is a likely consequence of its higher proliferation potential.

The role of VRK1 in controlling several aspects of cell proliferation [16], and its implication in p53 and DNA-damage responses suggested that it might be a potential target for specific drug development [17, 33]. Targeting VRK1 kinase was initially proposed based on observation of p53 mediated responses [13, 33], a regulatory role that is altered in tumors with p53 mutations [24] by a mechanism that requires p53-induction of the autophagic pathway [14]. More recently, it has been proposed that since VRK1 is a complementary proliferation pathway, its targeting might be less deleterious for cells that direct targeting of the main mitotic pathway [37], and thus opening a potentially useful therapeutic window that needs to be characterized.

VRK1, like the other two members of the family, has a kinase domain with some structural differences with respect to the typical kinase-domain [38, 39]. These structural differences led to the proposal that VRK proteins are likely have a low sensitivity to most inhibitors, and thus specific inhibitors will have little cross reactivity with other kinases, thus VRK proteins have a low promiscuity index [40, 41]. Their low sensitivity to currently used kinase inhibitors supports this idea [40, 42, 43]. Development of specific inhibitors for VRK1 is highly likely and they might be very useful for cancer treatment in two ways. In one way, VRK1 can interfere with cell cycle progression and division and thus prevent, or reduce, tumor growth and expansion [16]. On the other way, inhibiting VRK1 role in DNA damage response to ionizing radiation or chemotherapy [21] can make tumor cells more sensitive to these treatments, and might consequently permit a reduction of toxic doses in new protocols based on combination therapies, once VRK1 inhibitors become available in the future. This role of VRK1 in DNA-damage response is in agreement with the predictive role of 53BP1 and BRCA1 expression in breast cancer [44, 45].

Breast tumors expressing estrogen receptor, but not ERBB2, more frequently give bone metastasis [46]. This is consistent with high VRK1 levels that contribute to cell proliferation and drug resistance, and also with high levels of VRK2 in these tumors [28, 29], which can facilitate cell invasion by regulation of Cox2 gene expression [47] and protection against apoptosis [48].

In conclusion high VRK1 levels can contribute to a poorer prognosis in breast cancer. The level of VRK1 protein determines the sensitivity of breast cancer cells to DNA-damage based treatments, such as ionizing radiation or doxorubicin. High VRK1 protein levels confer a stronger resistance to treatment, which is consistent with a poorer prognosis. This VRK1 contribution to make cells more resistant to DNA-damage based therapies and by facilitating tumor growth and probably dissemination, an effect that will be further facilitated if the tumor has p53 mutations. The underlying molecular base of this VRK1 biological effects suggest that a similar situation is very likely to occur in other types of cancers and is probably not restricted to breast cancer.

## MATERIAL AND METHODS

### Breast cancer and immunohistochemistry in tissue arrays

Biopsies from human breast cancer were provided by the National Tumor Bank at CIC-Hospital Universitario de Salamanca. Biopsies were prepared as previously reported [24]. Sections were counterstained with hematoxylin. VRK1 was detected with a rabbit polyclonal antibody [49]. For analysis of VRK1, estrogen and progesterone receptors and ErbB2 consecutive sections were used. ErbB2 or other markers were scored in a scale from 1 to 3, regarding both intensity of staining and the number of positive cells, and the final score was determined by multiplying the two components [28]. Values were compared either by the chi-square or the Fisher's exact test (SPSS program version 18; SPSS, Chicago), and differences with a P < 0.05 were considered statistically significant [23, 24]. Clinical data was not available for analysis in this series.

### Antibodies

In immunohistochemistry, nuclear VRK1 was detected with rabbit polyclonal antibody VC1 [26] or HPA000660 from Sigma; the cytosolic subpopulation was detected with murine monoclonal antibody 1F6 [26]. Human VRK1 was detected in immunofluorescences and western blots with monoclonal 1B5 [26] or polyclonal anti-VRK1 from Sigma (HPA000660). Antibodies 1B5 and anti-VRK1 (Sigma) were used in western blots at dilution 1:1000 and in immunofluorescences at 1:50 or 1:100 respectively. 53BP1 was detected in immunofluorescences with either polyclonal 53BP1 (H-300) (Santa Cruz Biotechnology) or monoclonal anti-53BP1 from Upstate at 1:50 dilution (no differences in foci number or size were found between the two antibodies detecting 53BP1). Anti-ER (SP1, rabbit polyclonal antibody from Master Diagnostic, Granada, Spain). Anti-HER2/ErbB2 (44E7 from Cell Signaling); anti-pErk (E-4); anti-Erk2 (C-14); anti-p53 (Pab 1801 and DO-1) all from Santa Cruz Biotechnology (Santa Cruz, CA). β-actin was detected with monoclonal antibody AC15 (Sigma, St. Louis, MO). The secondary antibodies used for immunoblots: anti-(mouse-HRP) and anti-(rabbit-HRP) were from GE Healthcare. Bands were visualized with an ECL kit (GE Healthcare) followed by exposure to X-Ray films (Fujifilm). The secondary antibodies used for immunofluorescences were FluoroLinkCy2 anti-mouse, FluoroLinkCy2 anti-rabbit, FluoroLinkCy3 anti-mouse and FluoroLinkCy3 anti-rabbit from GE Healthcare.

### Cell lines, transfections and immunoblots

Breast cancer cell lines, MCF7 and MDA-MB-231, were obtained from the ATCC, and their characterization in the context of breast cancer phenotypes has been published [5, 30]. MDA-MB-231 and MCF7 breast cancer cells, [50] were grown in DMEM with 10% fetal bovine serum. The methods for cell lysates and conditions for western blot have been reported [49, 51, 52]. Several additional cell lines were used including luminal and basal types. Luminal breast cancer cell lines: BT474, T47D and ZR75B were grown in RPMI. Basal Breast cancer cell lines: BT549 (basal B) and HCC1569 (basal A) were grown in RPMI; HS587T (basal B), MDA-MB-231 (basal B) were grown in DMEM. Their molecular characteristics have been reported [30].

### DNA damage

DNA damage was induced by cell irradiation with 3 Gy using a Gammacell 1000 Elite irradiator (Theratronics, Ottawa, Canada) with a ^137^Cs source. Cells were treated with doxorubicin (Sigma, St. Louis, MO) for the time and dose indicated in individual experiments.

### VRK1 knockdown

Specific silencing of VRK1 was performed using siVRK1-02 (siV1-02), designed with the SMARTselection algorithm (Dharmacon, Lafayette, CO) and obtained from Dharmacon (DHARMACON RNA Technologies). The sequence target of siVRK1-02 is 5'-CAAGGAACCTGGTGTTGAA-3'. As negative control, the “ON-TARGETplus siCONTROL Non-targeting siRNA” from DHARMACON was used. The efficiency of RNAi transfection was determined with “siGLO RISC-free siRNA” (DHARMACON) labeled with a red fluorochrome [21].

Cells were transfected with the indicated siRNA at a concentration of 20 nM using Lipofectamine 2000 Reagent” (Invitrogen, Carlsbad, CA) according to manufactures instructions. After transfection, cells were processed for specific experiments as previously reported [14, 17, 24].

### Immunofluorescence and confocal microscopy

MCF7 and MDA-MB-231 cells were grown on uncoated glass coverslips, placed in 60-mm plates. Cells were prepared as previously reported [49]. Fluorescence images were captured with a LEICA TCS SP5 DMI-6000B confocal microscope (Leica), using the following lasers: Argon (488 nm), DPSS (561 nm) and UV Diode (405 nm). Fluorescence images were analyzed with LEICA LAS AF (Leica) and ImageJ (NIH, http://rsb.info.nih.gov/ij) software.

### Competing interests

The authors have declared that no competing interests exist.

### Authors contributions

M.S. and M. S-G. designed and performed experiments on DNA-damage response. A.V., S.B. and I.F.F. designed and performed experiments and analysis of human breast cancers. M. V-C. designed and performed studies in human breast cancer cell lines. P.A.L. designed and coordinated the project and wrote the manuscript.

## SUPPLEMENTARY FIGURES


