# Supplementary material for: Vaccinia-related kinase 1 (VRK1) confers resistance to DNA-damaging agents in human breast cancer by affecting DNA damage response
Source: Oncotarget. 2014 Jan 17;5(7):1770–8. doi: 10.18632/oncotarget.1678 (PMC4039124; doi:10.18632/oncotarget.1678)
Supplement: Supplementary file 1 [file oncotarget-05-1770-s001.pdf]

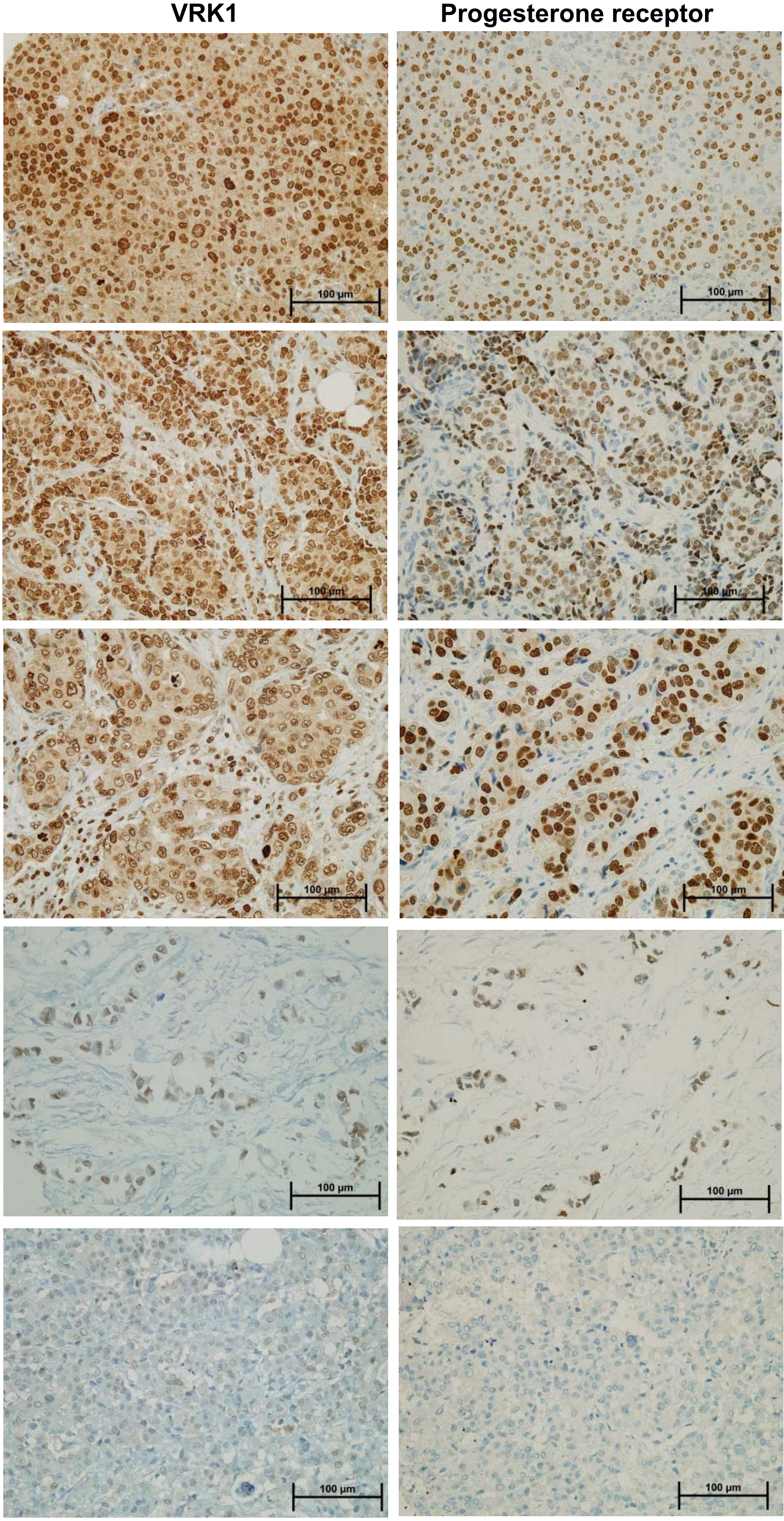

Figure S1. Positive correlation between VRK1 (left) and progesterone receptor (right) in breast cancer. Several breast cancer cases with different levels of VRK1 expression are shown with their corresponding levels of progesterone receptor. VRK1 was detected with rabbit polyclonal VC1antibody. Bar represents 100µm.

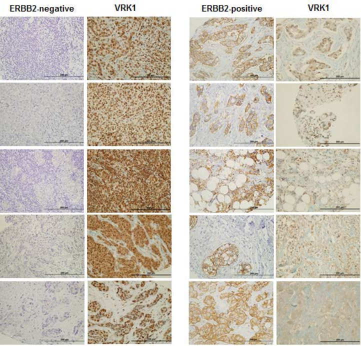

Figure S2. Inverse correlation between ERBB2 and VRK1 levels in breast cancer. In the left are shown five cases that are ERBB2 negative. To the right are shown five cases that are ERBB2 positive. VRK1 was detected with rabbit polyclonal VC1antibody. Bar represents 200 μm.

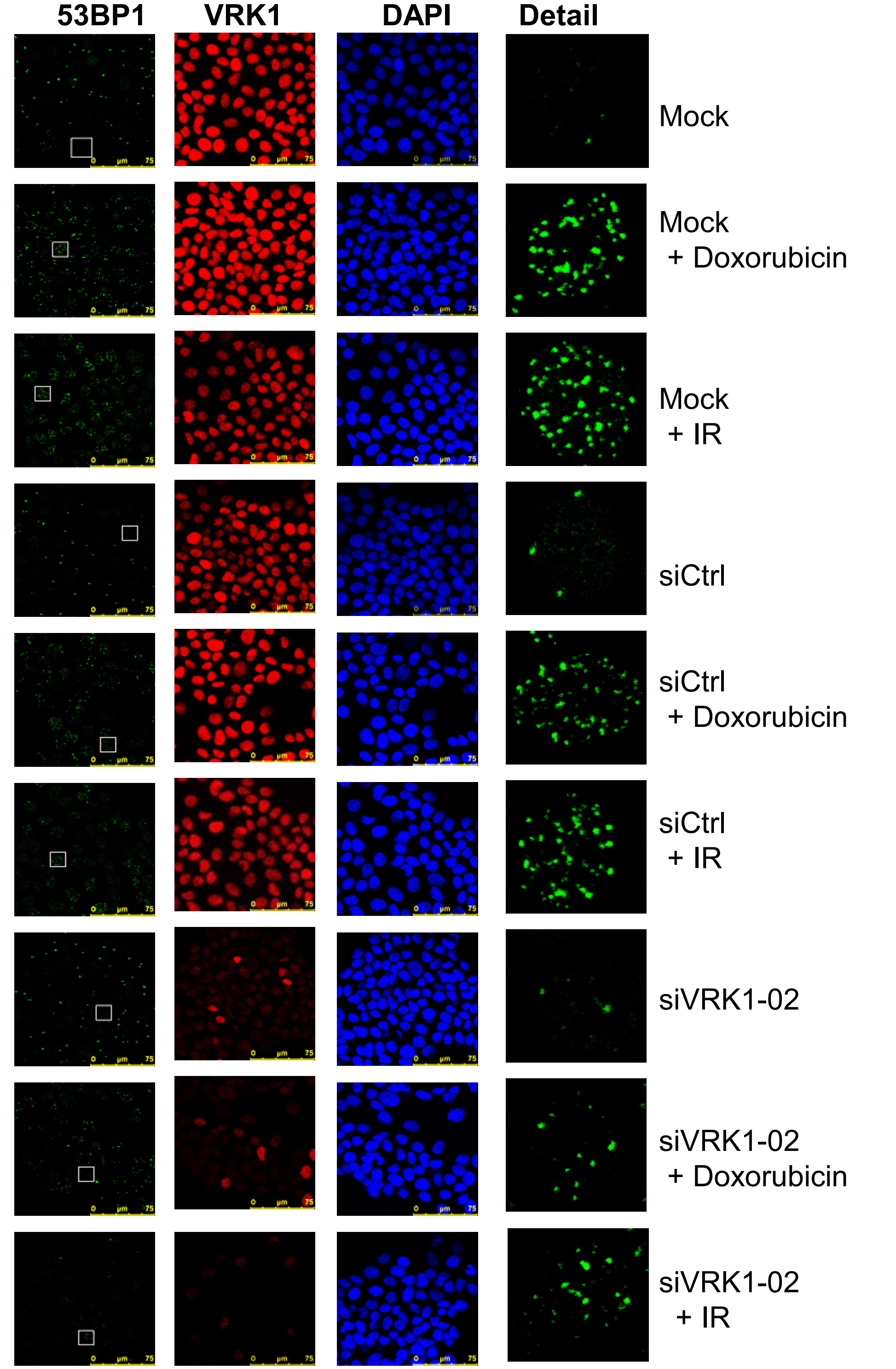

Figure S3. Effect of VRK1 knockdown on 53BP1 foci induced by ionizing radiation (IR) or doxorubicin in MCF7 breast cancer cells. At the top is indicated the protein detected. The column to the right shows detail of a single cell in the field. To the right is indiacted the interfering RNA used, control or VRK1-specific and the treatment (ionizing radiation or doxorubicin. Several field images from two experiments were used to quantify 53BP1 foci and are shown in Fig. 4.

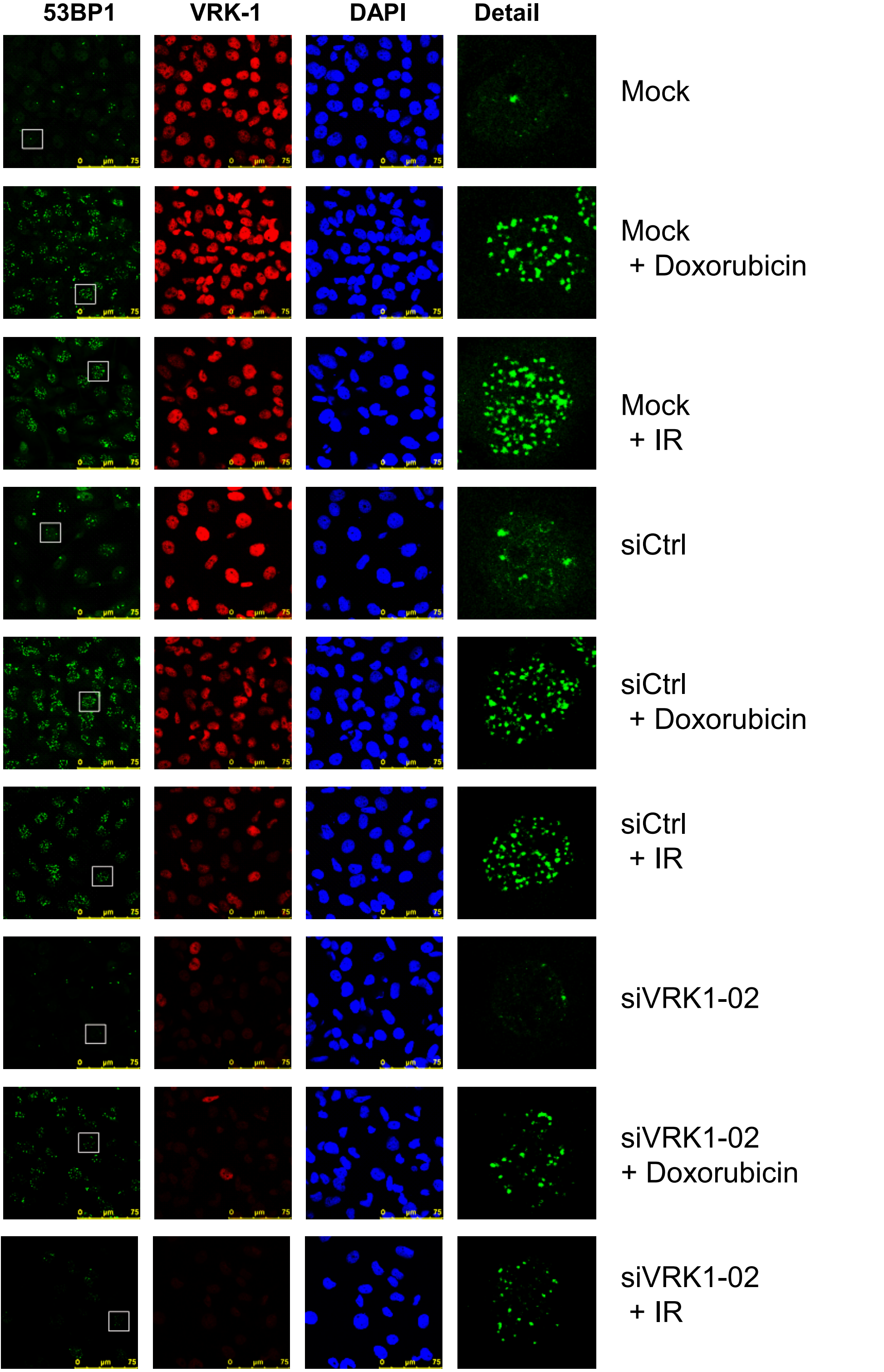

Figure S4. Effect of VRK1 knockdown of 53BP1 foci induced by ionizing radiation (IR) or doxorubicin in MDA-MB-231 breast cancer cells. At the top is indicated the protein detected. The column to the right shows detail of a single cell in the field. To the right is indiacted the interfering RNA used, control or VRK1-specific and the treatment (ionizing radiation or doxorubicin. Several field images from two experiments were used to quantify 53BP1 foci and shown in Fig. 5.
